# Conditioned Media from Adipose-Tissue-Derived Mesenchymal Stem Cells Downregulate Degradative Mediators Induced by Interleukin-1**β** in Osteoarthritic Chondrocytes

**DOI:** 10.1155/2013/357014

**Published:** 2013-12-10

**Authors:** Julia Platas, Maria Isabel Guillén, María Dolores Pérez del Caz, Francisco Gomar, Vicente Mirabet, Maria José Alcaraz

**Affiliations:** ^1^Department of Pharmacology, University of Valencia, Avenue Vicent Andrés Estellés s/n, Burjassot, 46100 Valencia, Spain; ^2^Department of Pharmacy, Cardenal Herrera CEU University, Moncada, 46113 Valencia, Spain; ^3^Department of Burn and Plastic Surgery, La Fe University Hospital, 46026 Valencia, Spain; ^4^Department of Surgery, Faculty of Medicine, University of Valencia, 46010 Valencia, Spain; ^5^Valencia Transfusion Center, Generalitat Valenciana, 46014 Valencia, Spain

## Abstract

Osteoarthritis (OA) is the most frequent joint disorder and an important cause of disability. Recent studies have shown the potential of adipose-tissue-derived mesenchymal stem cells (AD-MSC) for cartilage repair. We have investigated whether conditioned medium from AD-MSC (CM) may regulate in OA chondrocytes a number of key mediators involved in cartilage degeneration. CM enhanced type II collagen expression in OA chondrocytes while decreasing matrix metalloproteinase (MMP) activity in cell supernatants as well as the levels of MMP-3 and MMP-13 proteins and mRNA in OA chondrocytes stimulated with interleukin- (IL-) 1**β**. In addition, CM increased IL-10 levels and counteracted the stimulating effects of IL-1**β** on the production of tumor necrosis factor-**α**, IL-6, prostaglandin E_2_, and NO measured as nitrite and the mRNA expression of these cytokines, CCL-2, CCL-3, CCL-4, CCL-5, CCL-8, CCL-19, CCL-20, CXCL-1, CXCL-2, CXCL-3, CXCL-5, CXCL-8, cyclooxygenase-2, microsomal prostaglandin E synthase-1, and inducible NO synthase. These effects may be dependent on the inhibition of nuclear factor-**κ**B activation by CM. Our data demonstrate the chondroprotective actions of CM and provide support for further studies of this approach in joint disease.

## 1. Introduction

Osteoarthritis (OA) is a leading cause of disability in the elderly and has a significant impact on health care (reviewed in [1]). Although the pathogenesis of OA remains unclear, the chronic production of different mediators by articular tissues is believed to contribute to tissue degradation. Levels of proinflammatory cytokines such as interleukin- (IL-) 1*β* and tumor necrosis factor-*α* (TNF*α*) are elevated in the inflamed synovium in OA, accompanied by the increased expression of their receptors and decreased levels of inhibitory proteins. These cytokines mediate cartilage destruction through the upregulation of inflammatory or catabolic genes and the downregulation of anti-inflammatory or anabolic genes in articular chondrocytes (reviewed in [2]). In particular, IL-1*β* reduces the expression of type II collagen [3] and increases the production of matrix metalloproteinases (MMPs) [4, 5], prostaglandin E_2_ (PGE_2_), cytokines, chemokines, reactive oxygen species, and nitric oxide (NO) [6, 7]. Chondrocytes are the main source of NO in OA articular tissues and the oxidative stress caused by this mediator has been related to degeneration in arthritic joints [8]. Therefore, NO can play a role in IL-1*β*-induced suppression of glycosaminoglycan and collagen synthesis, expression of MMPs, and activation of proenzymes [9].

Mesenchymal stem cells (MSC) are being investigated as a possible cell-based therapy for late stages of OA [10]. Some promising results have been obtained in a pilot study of knee OA using autologous bone-marrow-derived mesenchymal stem cells [11]. Interestingly, stem cells are able to secrete a wide range of trophic mediators that can exert paracrine effects on other cell types. Therefore, stem-cell-conditioned media have shown potential therapeutic applications in neural, myocardial, and osteogenic regeneration or in wound healing (reviewed in [12]). Adipose-tissue-derived mesenchymal stem cells (AD-MSC) are extensively investigated for tissue regeneration or immunomodulation (reviewed in [13, 14]). Interestingly, stem cells are able to secrete a wide range of trophic mediators that can exert paracrine effects on other cell types. In this study, we have examined the potential of the conditioned medium from basal AD-MSC (CM) to regulate type II collagen expression and the production of relevant mediators involved in OA articular degeneration using OA chondrocytes in primary cultures as an in vitro model to study inflammatory and degradative responses [15].

## 2. Materials and Methods

### 2.1. Cells and Culture Media

Adipose tissues were obtained from 11 donors who had undergone abdominoplasty without any underlying diseases. Adipose tissue samples were washed with phosphate-buffered saline (PBS), minced, digested at 37°C for 1 h with 2% of type I collagenase (Gibco, Life Technologies, Madrid, Spain), and filtered through a 100 *μ*m cell strainer (BD Biosciences Durham, NC, USA). The cells were washed with DMEM/HAM F12 containing penicillin and streptomycin (1%), seeded onto tissue culture flasks in DMEM/HAM F12 medium with penicillin and streptomycin (1%) supplemented with 15% human serum, and incubated with 5% CO_2_ at 37°C. Human serum was obtained from whole-blood donations of AB-blood-group-typed donors according to the criteria of Valencia Transfusion Center. At 24 h, when the cells reached the semiconfluence, the tissue culture plates were washed to remove any residual nonadherent cells. The phenotype of AD-MSC was analyzed by flow cytometry (Flow Cytometer II, BD Biosciences, San Jose, CA, USA) with specific antibodies, anti-CD105-PE, anti-CD90PerCP-eFluo 710, anti-CD34APC (eBioscience, Inc., San Diego, CA, USA), and anti-CD45-PE (BD Pharmigen) and cellular viability with propidium iodide. CM was collected from cells at passages 0 and 1 every 48 h of culture, pooled, centrifuged, and stored at −80°C in sterile conditions.

The knee specimens were obtained from patients with the diagnosis of advanced OA (22 women and 8 men, aged 72.1 ± 7.8 years, mean ± S.E.M.) undergoing total knee joint replacement. Diagnosis was based on clinical, laboratory, and radiological evaluation. Cartilage was dissected from the femoral condyles and tibial plateau of the knee joint and diced into small pieces. Human articular chondrocytes were isolated by sequential enzymatic digestion: 1 h with 0.1 mg/mL hyaluronidase (Sigma-Aldrich) followed by 12–15 h with 2 mg/mL collagenase (type IA) (Sigma-Aldrich) in DMEM/HAM F12 (Sigma-Aldrich) containing penicillin and streptomycin (1%) at 37°C in 5% CO_2_ atmosphere. The digested tissue was filtered through a 70 *μ*m nylon mesh (BD Biosciences), washed, and centrifuged. Cell viability was greater than 95% according to the Trypan blue exclusion test. All experiments were performed with chondrocytes in primary cultures at semiconfluence (270 × 10^3^ cells/well in 6-well plates or 1.5 × 10^6^ cells in 3.5 cm plates). Chondrocytes were maintained with 5% CO_2_ at 37°C in DMEM/HAM F12 (Sigma-Aldrich) containing penicillin and streptomycin (1%), supplemented with 10% fetal bovine serum (Sigma-Aldrich). For cell stimulation, chondrocytes were incubated for 24 h or 5 days in DMEM/HAM F12 (Sigma-Aldrich) containing penicillin and streptomycin (1%) supplemented with 10% human serum, in the presence or absence of IL-1*β* (10 ng/mL) and/or CM (1 mL of medium for 6-well plates or 2 mL for 3.5 cm plates).

The design of the work was approved by the Institutional Ethical Committees (University of Valencia and University Clinical Hospital, Valencia, Spain). Samples were obtained from donors after they provided informed consent according to the Helsinki Declaration of 1975, as revised in 2008.

### 2.2. MTT Assay

The mitochondrial dependent reduction of 3-(4,5-dimethylthiazol-2-yl)-2,5 diphenyltetrazolium bromide (MTT) to formazan was assayed in OA chondrocytes incubated with IL-1*β* (10 ng/mL) or IL-1*β* (10 ng/mL) + CM for 24 h or 5 days. The cells were then incubated with MTT (200 *μ*g/mL) for 2 h. The medium was removed and the cells were solubilized in dimethyl sulfoxide (100 *μ*L) to quantitate formazan at 550 nm [16].

### 2.3. Immunocytochemistry

Chondrocytes were seeded at 20 × 10^3^ cells/well in Lab-tek chambers (Thermo Scientific, Rochester, NY, USA) and stimulated with IL-1*β* (10 ng/mL) or IL-1*β*+CM for 5 days. Cells were fixed with 4% formaldehyde in PBS for 30 min at 4°C and incubated with rabbit anti-human type II collagen polyclonal antibody (Chemicon/Millipore, Schwalbach, Germany). Finally, chondrocytes were incubated with goat anti-rabbit IgG-FITC (R&D Biosystems, Abingdon, UK). Slides were mounted in Prolong Gold antifade reagent with DAPI (Molecular Probes, Invitrogen, Life Technologies) and examined under a fluorescence microscope (Leica DM IL LED, Solms, Germany). Cells were counted in 6 microscopic fields of each well. Thereby collagen II-positive cells were determined as a percentage of total cell number.

### 2.4. Determination of MMP Activity

Chondrocytes were stimulated with IL-1*β* (10 ng/mL) or IL-1*β*+CM for 24 h or 5 days and supernatants were harvested, centrifuged, and incubated with *p*-aminophenylmercuric acetate for 12 h at 37°C to activate MMPs. Aliquots of supernatants were then transferred to a 96-well plate and after addition of the 5-FAM peptide substrate (AnaSpec Inc., San Jose, CA, USA), fluorescence was measured for different times at 490 nm (excitation)/520 nm (emission) in a Victor3 microplate reader (PerkinElmer España, Madrid, Spain).

### 2.5. Enzyme-Linked Immunosorbent Assay

Chondrocytes were stimulated with IL-1*β* (10 ng/mL) or IL-1*β*+CM for 24 h or 5 days. Supernatants were harvested, centrifuged, and frozen at −80°C until analysis. TNF*α*, IL-6, and IL-10 were measured by enzyme-linked immunosorbent assay (ELISA) kits from eBioscience (San Diego, CA, USA) with sensitivity of 4.0 pg/mL for TNF*α* and IL-6 and 2.0 pg/mL for IL-10. MMP-3 and MMP-13 proteins were measured by ELISA kits (eBioscience) with sensitivity of 8.0 and 18.0 pg/mL, respectively. Nuclear factor-*κ*B (NF-*κ*B) binding to DNA was quantified by ELISA in nuclear extracts from cells stimulated with IL-1*β* (10 ng/mL) in the presence or absence of CM for 1 h, using the Nuclear Extract Kit Active Motif for nuclei extraction followed by TransAM NF-*κ*B kit (Active Motif Europe, Rixensart, Belgium), according to the manufacturer's recommendations.

### 2.6. Real-Time PCR

Total RNA was extracted using the TriPure reagent (Roche Applied Science, Barcelona, Spain) according to the manufacturer's instructions. Reverse transcription was accomplished on 1 *μ*g of total RNA using random primers (TaqMan reverse transcription reagents, Applied Biosystems, Madrid, Spain). PCR assays were performed in duplicate on an iCycler Real-Time PCR Detection System using SYBR Green PCR Master Mix (Bio-Rad Laboratories, Richmond, CA, USA). Sequences of primers used have been reported previously [17–21] and were synthesized by Eurofins MWG Operon (Ebersberg, Germany). Some primer sets were from SA Biosciences Corporation (Tebu-Bio, Barcelona, Spain). For each sample, differences in threshold cycle (ΔCt) values were calculated by correcting the Ct of the gene of interest to the Ct of the reference gene *β*-actin. Relative gene expression was expressed as 2^−ΔΔCt^ with respect to nonstimulated cells.

### 2.7. Determination of NO and PGE_2_

Chondrocytes were stimulated with IL-1*β* (10 ng/mL) or IL-1*β*+CM for 24 h or 5 days. Supernatants were used to measure prostaglandin E_2_ (PGE_2_) by radioimmunoassay [22] and nitrite by a fluorometric method [23] using a Victor3 microplate reader (PerkinElmer España, Madrid, Spain).

### 2.8. Statistical Analysis

The data were analyzed by one-way analysis of variance (ANOVA) followed by Bonferroni's posttest using the GraphPad Prism 5 software (GraphPad Software, La Jolla, CA, USA). A *P* value of less than 0.05 was considered to be significant.

## 3. Results

### 3.1. Cell Proliferation

**Figure 1:**
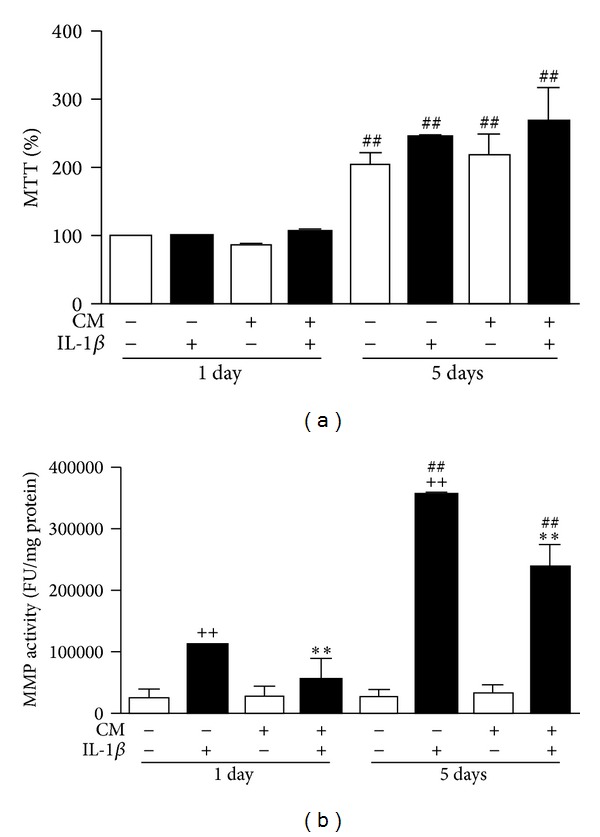
Effects of CM on (a) cell proliferation and (b) MMP activity in OA chondrocytes. (a) Cell proliferation was measured by the MTT method. Results are expressed as % MTT with respect to nonstimulated cells at 24 h. (b) MMP activity was measured by fluorometry in cell culture supernatants after 24 h or 5 days of incubation of chondrocytes in the presence or absence of IL-1*β* and CM. Activity is expressed as fluorescence units (FU) per mg of protein. Data represent mean ± S.E.M. of independent cultures with chondrocytes from 4 different donors. ^++^ *P* < 0.01 with respect to nonstimulated cells; **P < 0.01 with respect to IL-1*β*; ^##^ *P* < 0.01 with respect to the corresponding group after 24 h incubation.

To determine whether CM treatment affected OA chondrocyte proliferation, OA chondrocytes were incubated in the absence or presence of CM and IL-1*β*. After 24 h or 5 days, the MTT reaction was performed. As shown in Figure 1(a), a significantly higher MTT % was observed for all groups after 5 days with respect to 24 h incubation. The presence of CM did not induce any significant changes in cell proliferation.

### 3.2. Effects on Matrix Metalloproteinases

**Figure 2:**
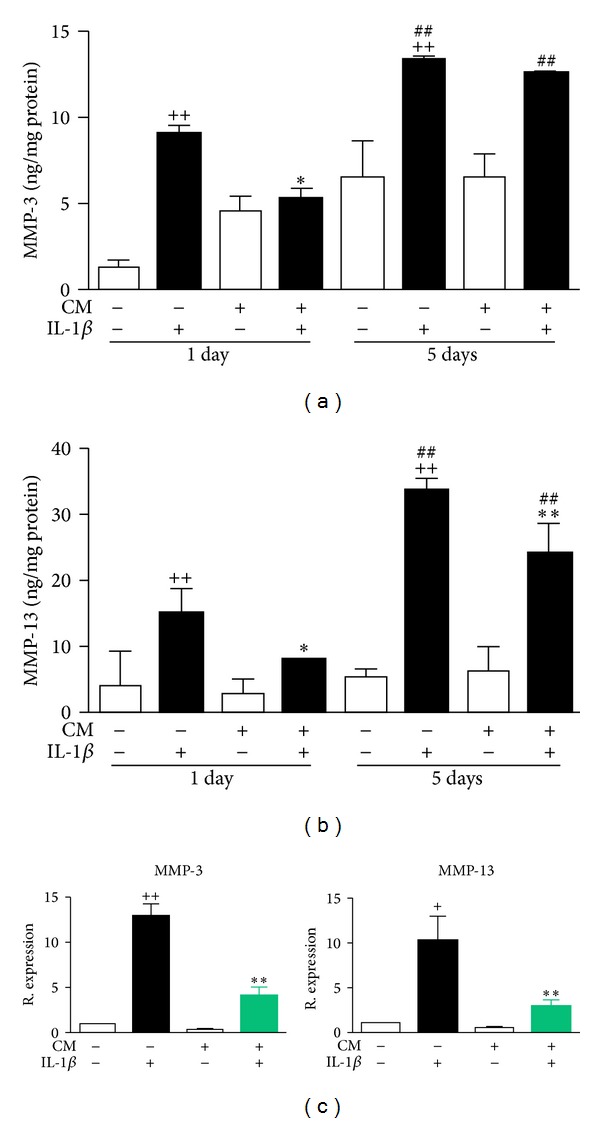
Effects of CM on MMP-3 (a) and MMP-13 (b) proteins and mRNA (c) expression in OA chondrocytes. (a, b) Protein levels were measured by ELISA in cell supernatants after 24 h or 5 days of incubation of chondrocytes in the presence or absence of IL-1*β* and CM. (c) mRNA expression was determined by real-time PCR analysis in OA chondrocytes after 24 h of incubation in the presence or absence of IL-1*β* and CM. Results indicate relative expression with respect to nonstimulated OA chondrocytes. Data represent mean ± S.E.M. of independent cultures with chondrocytes from 4 different donors. ^+^ *P* < 0.05, ^++^ *P* < 0.01 with respect to nonstimulated cells; *P < 0.05, **P < 0.01 with respect to IL-1*β*; ^##^ *P* < 0.01 with respect to the corresponding group after 24 h incubation.

MMPs are key mediators in cartilage degradation. To characterize the effects of CM on OA chondrocytes, we measured MMP activity as well as the levels of MMP-3 and MMP-13 proteins in cell supernatants. In addition, we determined mRNA expression in OA chondrocytes. IL-1*β* enhanced the MMP activity present in cell supernatants after 24 h of incubation and to a greater extent after 5 days (Figure 1(b)). Interestingly, MMP activity was significantly reduced by CM at both time points. MMP-3 protein (Figure 2(a)) and mRNA (Figure 2(c)) levels were decreased by CM in IL-1*β*-stimulated chondrocytes and this effect was significant after 24 h of incubation.  Figure 2(b) shows that MMP-13 protein levels were reduced by CM after IL-1*β* stimulation (24 h and 5 days of incubation). MMP-13 mRNA expression was also significantly reduced by CM in IL-1*β*-stimulated OA chondrocytes (Figure 2(c)).

### 3.3. Effects on Collagen II Expression

**Figure 3:**
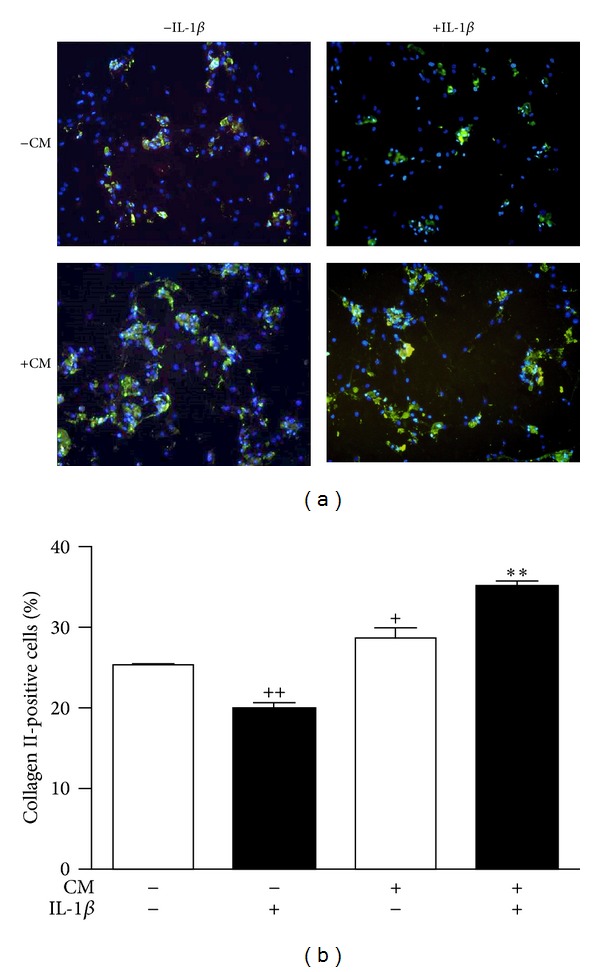
Immunocytochemical analysis of collagen II expression. Chondrocytes were treated with CM and/or IL-1*β* for 5 days. (a) Representative image showing the expression of collagen II (FITC-immunofluorescence, green) and DAPI (blue) for cellular nuclei. Original magnification ×200. (b) Percentages of collagen II-positive cells with respect to total cell numbers. Data represent mean ± S.E.M. of independent cultures with chondrocytes from 4 different donors. ^+^ *P* < 0.05, ^++^ *P* < 0.01 with respect to nonstimulated cells; **P < 0.01 with respect to IL-1*β*.

Collagen II is a marker of articular chondrocyte functionality.  Figure 3(a) shows a representative image of collagen II expression in OA chondrocytes in the presence or absence of IL-1*β* and CM after 5 days of incubation. This cytokine reduced the expression of collagen II, but CM significantly increased the percentage of collagen II-positive cells either in basal conditions or in chondrocytes stimulated with IL-1*β* (Figure 3(b)).

### 3.4. Effects on Cytokines and Chemokines

**Figure 4:**
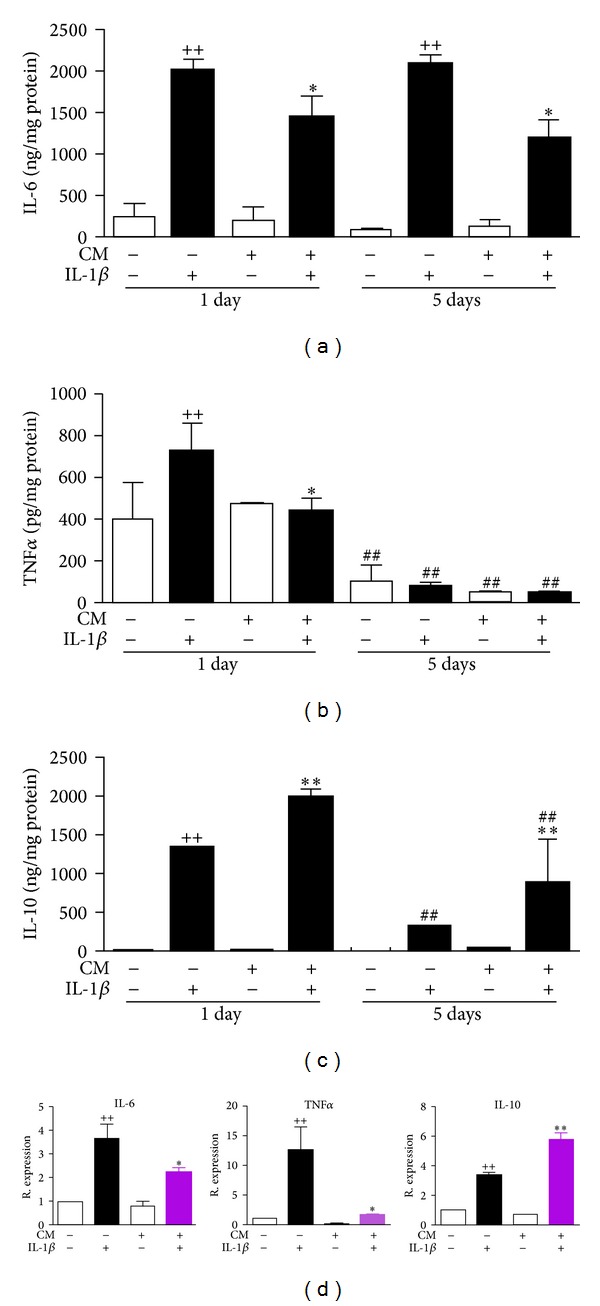
Effects of CM on the protein levels of IL-6 (a), TNF*α* (b), and IL-10 (c) in supernatants and mRNA expression in OA chondrocytes (d). (a–c) Cytokines were measured by ELISA in cell culture supernatants after 24 h or 5 days of incubation of chondrocytes with CM in the presence or absence of IL-1*β*. (d) mRNA expression was determined by real-time PCR analysis in OA chondrocytes after 24 h of incubation and results indicate relative expression with respect to nonstimulated OA chondrocytes. Data represent mean ± S.E.M. of independent cultures with chondrocytes from 6 different donors. ^++^ *P* < 0.01 with respect to nonstimulated cells; *P < 0.05, **P < 0.01 with respect to IL-1*β*; ^##^ *P* < 0.01 with respect to the corresponding group after 24 h incubation.

**Figure 5:**
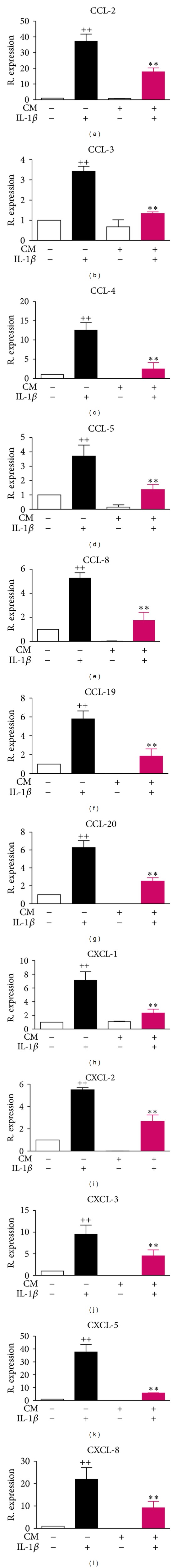
Effects of CM on chemokine mRNA expression. mRNA expression was determined by real-time PCR analysis in OA chondrocytes after 24 h of incubation in the presence or absence of IL-1*β* and/or CM. Results indicate relative expression with respect to nonstimulated OA chondrocytes. Data represent mean ± S.E.M. of independent cultures with chondrocytes from 4 different donors. ^++^ *P* < 0.01 with respect to nonstimulated cells; **P < 0.01 with respect to IL-1*β*.

IL-6 and TNF*α* are key mediators of the inflammatory response and were measured in supernatants by ELISA.  Figure 4(a) shows that IL-1*β* strongly increased IL-6 levels in cell supernatants after 24 h or 5 days of incubation whereas CM significantly decreased the production of IL-6 at both time points. In addition, the levels of TNF*α* induced by IL-1*β* were reduced by CM after 24 h incubation (Figure 4(b)). Interestingly, the production of the anti-inflammatory cytokine IL-10 was significantly enhanced by CM at both time points (Figure 4(c)). The results on mRNA expression paralleled the effects of CM on protein levels. Therefore, we observed a reduced expression of IL-6 and TNF*α* while IL-10 mRNA expression was significantly enhanced by CM in IL-1*β*-stimulated chondrocytes (Figure 4(d)). In OA, there is an increased expression of chemokines in chondrocytes under the influence of inflammatory cytokines such as IL-1*β* [24, 25].  Figure 5 shows that IL-1*β* enhanced the mRNA expression of CCL-2, CCL-3, CCL-4, CCL-5, CCL-8, CCL-19, CCL-20, CXCL-1, CXCL-2, CXCL-3, CXCL-5, and CXCL-8 after 24 h of incubation. When CM was included in the incubation media, we observed a significant reduction in the expression of these chemokines.

### 3.5. Production of NO and PGE_2_

**Figure 6:**
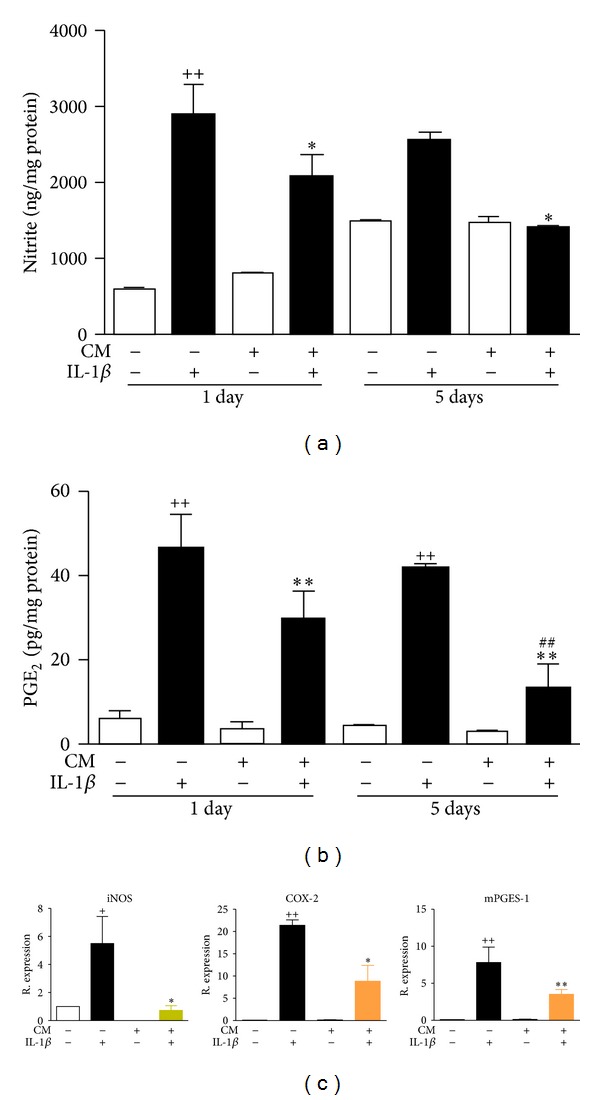
Effects of CM on NO (a) and PGE_2_ (b) production and iNOS, COX-2, and mPGES-1 mRNA (c) expression in OA chondrocytes. (a) NO was measured by fluorometry as nitrite. (b) PGE_2_ was measured by radioimmunoassay. Mediators were determined in cell culture supernatants after 24 h or 5 days of incubation of OA chondrocytes with CM in the presence or absence of IL-1*β*. (c) mRNA expression was determined by real-time PCR analysis in OA chondrocytes after 24 h of incubation and results indicate relative expression with respect to nonstimulated OA chondrocytes. Data represent mean ± S.E.M of independent cultures with chondrocytes from 6 (NO, PGE_2_) or 4 (mRNA) different donors. ^++^ *P* < 0.01 with respect to nonstimulated cells; *P < 0.05, **P < 0.01 with respect to IL-1*β*; ^##^ *P* < 0.01 with respect to the corresponding group after 24 h incubation.

We also investigated the production of other relevant mediators in our experimental system. The concentrations of nitrite, as an index of NO production, and PGE_2_were measured in the supernatant of OA chondrocytes after 24 h or 5 days of incubation. The presence of CM during the incubation period resulted in a significant reduction in the levels of nitrite (Figure 6(a)) and PGE_2_ (Figure 6(b)) in the supernatant at both time points in the presence of IL-1*β* stimulation. We analyzed the relative mRNA expression of inducible NO synthase (iNOS), cyclooxygenase-2 (COX-2), and microsomal PGE synthase-1 (mPGES-1) in OA chondrocytes incubated with IL-1*β* and/or CM for 24 h.  Figure 6(c) shows that CM significantly reduced the expression of iNOS thus leading to reduced nitrite production, in addition to the downregulation of COX-2 and mPGES-1 which would explain the inhibitory effects of CM on PGE_2_.

### 3.6. NF-*κ*B Activation

**Figure 7:**
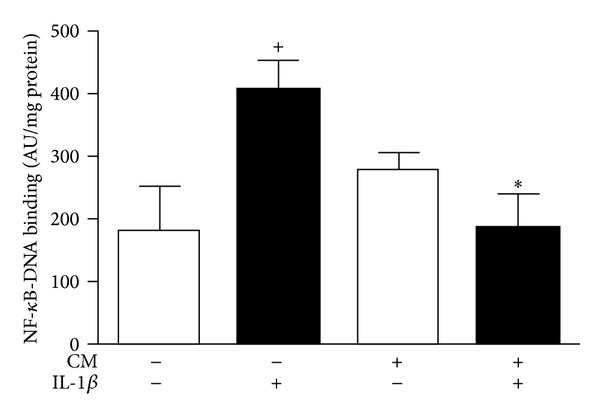
Effects of CM on NF-*κ*B activation in OA chondrocytes. Cells were stimulated with IL-1*β* for 1 h and p65-NF-*κ*B binding to DNA was determined by ELISA in nuclear fractions. Data are expressed as mean ± S.E.M. of independent cultures with chondrocytes from 3 different donors. AU: arbitrary units. ^+^ *P* < 0.05 with respect to nonstimulated cells; *P < 0.05 with respect to IL-1*β*.

To understand the mechanism involved in the effects of CM on inflammatory and catabolic mediators, we investigated the possible regulation of this key transcription factor. IL-1*β* quickly induces NF-*κ*B translocation into the nucleus and DNA binding to activate gene transcription.  Figure 7 shows the enhancement of p65-NF-*κ*B-DNA binding induced by IL-1*β* in OA chondrocytes. In the presence of CM, p65-NF-*κ*B binding to DNA was significantly decreased.

## 4. Discussion

Several lines of evidence have demonstrated in OA articular tissues the production of a wide range of catabolic and proinflammatory mediators leading to cartilage matrix degradation (reviewed in [26]). In addition to other extracellular matrix components, the fibrils of type II collagen are essential for the integrity and survival of articular cartilage. In OA cartilage, there is an upregulation of collagenases such as MMP-13, the major type II collagen-degrading enzyme, which initiate the denaturation of fibrillar type II collagen thus contributing to the initiation and progression of joint damage [27]. In this study, we have demonstrated that CM enhances collagen II expression in nonstimulated OA chondrocytes and counteracts the negative effects of IL-1*β* on this protein. We have also found that CM downregulates catabolic enzymes which play a key role in cartilage degradation. Therefore, CM reduced the production of the collagenase MMP-13 and to a lesser extent of the stromelysin MMP-3 which mediates the direct degradation of extracellular matrix components and the activation of other MMPs [28].

Proinflammatory cytokines activate chondrocytes leading to the synthesis and release of a wide range of mediators. Our study reveals that CM downregulates the proinflammatory cytokines IL-6 and TNF*α* but upregulates the anti-inflammatory cytokine IL-10. These effects are of relevance for the progression of OA as proinflammatory cytokines induce the synthesis of molecules contributing to the loss of chondrocyte phenotype and cartilage degeneration [2]. In addition, the effect of CM on IL-10 may have positive consequences on cartilage metabolism as this cytokine cooperates with other factors to inhibit cartilage breakdown in experimental OA [29].

Chondrocytes may amplify inflammatory and catabolic responses through the release of chemokines promoting inflammation, synovial angiogenesis [30, 31], and the production of catabolic mediators such as MMPs in OA chondrocytes [32, 33]. Interestingly, CM reduced the expression of a number of chemokines relevant to chondrocyte metabolism. Our data also show that CM counteracts NO production in OA chondrocytes stimulated by IL-1*β*. This effect would be the consequence of iNOS downregulation which may contribute to the protective actions of CM as NO inhibits matrix synthesis [8]. In addition, we have shown the inhibitory effects of CM on PGE_2_ production. This eicosanoid may contribute to cartilage degradation by promoting the production of MMPs and inhibiting the synthesis of tissue inhibitors of these enzymes [34, 35]. COX-2 and mPGES-1 are functionally coupled and induced by IL-1*β* in OA chondrocytes leading to an increased PGE_2_ synthesis [36]. Therefore, the effect of CM on PGE_2_ production by OA chondrocytes may be relevant to cartilage metabolism and would be dependent on the reduction of COX-2 and mPGES-1 expression observed in our study.

The activation of NF-*κ*B plays a key role in the transcription of iNOS, COX-2, MMPs, and different proinflammatory cytokines and chemokines [37, 38]. To understand the possible mechanism by which CM downregulates these mediators, we have investigated its influence on NF-*κ*B. Our results indicate that the observed inhibitory effects of CM on the expression of catabolic and proinflammatory molecules could be related to the reduction of NF-*κ*B activation in OA chondrocytes stimulated with IL-1*β*.

MSC have shown cytoprotective properties in experimental models via paracrine mechanisms. The beneficial effects of these cells may be related to the production of different types of mediators such as cytokines and PGE_2_ or a milieu of secreted factors exerting synergistic effects [39–41]. Further studies are needed to elucidate the factors responsible for the chondroprotective effects of CM or to provide additional mechanistic insights.

## 5. Conclusion

The results of our study have shown the chondroprotective role of CM by targeting catabolic and inflammatory mediators and support the interest of this approach to search for new treatments for inflammatory and/or degenerative conditions of joints.

## Abbreviations

AD-MSC: Adipose tissue-derived mesenchymal stem cells
CM: AD-MSC conditioned medium
COX-2: Cyclooxygenase-2
ELISA: Enzyme-linked immunosorbent assay
IL: Interleukin
iNOS: Inducible nitric oxide synthase
MMP: Matrix metalloproteinase
mPGES-1: Microsomal prostaglandin E synthase-1
MSC: Mesenchymal stem cells
NF-*κ*B: Nuclear factor *κ*B
NO: Nitric oxide
OA: Osteoarthritis
PBS: Phosphate-buffered saline
PGE_2_: Prostaglandin E_2_
TNF*α*: Tumor necrosis factor-*α*.
